# Effectiveness of I-Exercises: A Comprehensive Ocular Exercise Protocol for Reducing Ocular Fatigue Among Students With Myopia in a Randomized Controlled Trial

**DOI:** 10.7759/cureus.115148

**Published:** 2026-08-25

**Authors:** Dhvani Shah, Bhavana R Gadhavi, P. Antony Leo Asser, Pothiraj Pitchai, Dipali Suvarna, Fatima M Thoufeek, Vanshika Desai, Rupali Shevalkar

**Affiliations:** 1 Department of Physiotherapy, Parul University, Vadodara, IND; 2 Department of Physiotherapy, Parul Institute of Physiotherapy, Parul University, Vadodara, IND; 3 Department of Physiotherapy, Sri Ramachandra Institute of Higher Education and Research, Chennai, IND; 4 Department of Physiotherapy, K J Somaiya College of Physiotherapy, Mumbai, IND

**Keywords:** control group, humans, prevalence, questionnaires, surveys

## Abstract

Background

The prevalence of ocular fatigue associated with prolonged digital device use and sustained near-vision tasks is increasing among students with myopia. However, evidence regarding the effectiveness of structured ocular exercise programs remains limited.

Objective

To determine the effectiveness of a Comprehensive Ocular Exercise Module (I-Exercises) in reducing ocular fatigue symptoms among students with myopia.

Methods

A randomized controlled trial was conducted among 40 participants aged 18-25 years with myopia. Participants were randomly allocated using a computer-generated sequence to an experimental group (n = 20) that received I-Exercises or a control group (n = 20) that received no intervention. Participants in the experimental group performed supervised ocular exercises five times per week for three weeks. Ocular fatigue was assessed before and after the intervention using a 12-item ocular fatigue questionnaire adapted from a previously published instrument. Each symptom was scored independently from 0 to 6, with higher scores indicating greater severity.

Results

Participants receiving I-Exercises demonstrated significant improvement in several ocular fatigue symptoms compared with the control group (p < 0.05). Significant between-group improvements were observed in tired eyes, sore/aching eyes, irritated eyes, watery eyes, eyestrain, blurred vision, difficulty focusing, and visual discomfort, whereas red eyes, dry eyes, hot/burning eyes, and double vision did not show statistically significant between-group differences.

Conclusion

The I-Exercises Module may help reduce selected ocular fatigue symptoms among students with myopia and may serve as a non-invasive approach for reducing symptoms associated with prolonged near-vision tasks. These findings should be interpreted as preliminary given the small sample size, short intervention period, and exploratory nature of the individual symptom-level analyses.

## Introduction

Vision is the most important sense; the majority (about 80%) of our sensory information comes from what we see [1]. With modern lifestyles increasingly reliant on digital devices, including computers and smartphones, as well as reading, there has been a substantial rise in near- to intermediate-vision activities that require sustained accommodation and convergence, involving continuous use of the extraocular and ciliary muscles [2]. The increased visual demand associated with these activities can lead to visual fatigue, commonly experienced as eye fatigue, dry eyes, hazy vision, difficulty focusing, ocular discomfort, and tired or aching eyes [3].

Students and young adults are particularly vulnerable due to increased screen use associated with educational and leisure activities, which has been linked to reduced concentration [4]. Recent trends indicate a growing prevalence of myopia (nearsightedness) among adolescents and young adults [5]. Uncorrected or inadequately managed refractive errors continue to be among the major causes of visual impairment worldwide and were a major focus of the World Health Organization's Vision 2020: The Right to Sight initiative [6]. Myopia, the predominant refractive error in this population, is influenced by environmental factors as well as genetic predisposition, including prolonged near-vision tasks and sustained visual stress [7].

Chronic visual strain may contribute to ocular fatigue, reducing visual comfort and potentially affecting academic performance and quality of life among individuals exposed to prolonged near work. Students with myopia may experience additional accommodative and visual stress associated with sustained near-vision demands. Although corrective lenses improve refractive status, they do not specifically address symptoms of visual fatigue associated with prolonged near work. Therefore, interventions targeting ocular function and visual endurance may be clinically relevant [8,9].

Eye exercises have been proposed as a non-invasive approach to reducing eye fatigue by improving visual coordination, endurance, and relaxation. Various approaches, including yogic eye exercises, have demonstrated potential benefits in reducing symptoms of eye fatigue [10,11]. Despite these findings, ocular physiotherapy remains under-researched, with limited standardized and evidence-based exercise protocols available [12].

Given the increasing incidence of myopia and digital eye strain among students, and the limited availability of structured ocular exercise protocols, there is a need to evaluate targeted ocular exercise interventions to reduce ocular fatigue. Therefore, the current investigation was conducted to determine the effectiveness of the Comprehensive Ocular Exercise Module (I-Exercises) in reducing self-reported ocular fatigue symptoms among students with myopia compared with a control group.

## Materials and methods

Study design

This prospective, randomized controlled trial was conducted to assess the effectiveness of a Comprehensive Ocular Exercise Module (I-Exercises) in reducing ocular fatigue among individuals with myopia. All participants provided written informed consent before enrollment, and the study was approved by the Institutional Ethics Committee. The trial was prospectively registered with the Clinical Trials Registry of India (CTRI) (Registration No.: CTRI/2024/11/076157).

The present manuscript reports the eye-fatigue component of the IEC-approved project, which evaluated the effects of the comprehensive ocular exercise module on visual evoked potential, eye fatigue, and visual acuity in individuals with myopia. Thus, the ocular-fatigue outcomes reported in the present study represent one of the prespecified outcome domains of the approved project. No separate protocol amendment was made specifically for the ocular-fatigue assessment.

Participants

A total of 40 participants diagnosed with myopia were included in the study. Randomization was performed using a computer-generated random number sequence. Participants were randomly allocated into two groups: an experimental group (n = 20) and a control group (n = 20) [13]. The randomization procedure was completed before commencement of the intervention. Due to the nature of the exercise intervention, blinding of participants and the treating physiotherapist was not feasible. As ocular fatigue was assessed using a self-reported questionnaire, the possibility of response and expectation bias could not be excluded.

Inclusion criteria

Participants included students of either gender aged 18-25 years with unilateral or bilateral myopia, including those with high myopia. To ensure refractive stability and minimize the inclusion of recently diagnosed or fluctuating refractive conditions, only individuals with a minimum duration of one year since the diagnosis of myopia were enrolled. All participants were required to be willing to participate in the study and provide written informed consent. As myopia had been diagnosed before enrollment, refractive-error grading was not performed as part of the present study and was not considered an outcome measure.

Exclusion criteria

Students were excluded if they had diagnosed systemic conditions such as diabetes mellitus or thyroid disorders, intellectual disability, a history of refractive surgery (e.g., LASIK), any pathological ocular condition or congenital eye abnormality, or any refractive error other than myopia.

Intervention

Participants in the experimental group received the Comprehensive Ocular Exercise Module (I-Exercises) [14]. The module had previously been developed and standardized using a Delphi consensus process involving expert consultation and evidence-based ocular exercise principles. The Delphi study established the content validity and feasibility of the protocol; however, its clinical effectiveness had not been evaluated. Therefore, the present randomized controlled trial was conducted to evaluate the effectiveness of the I-Exercises module in reducing ocular fatigue among students with myopia.

None of the participants reported using eye drops or other ocular medications during the study period. Participants allocated to the control group continued their usual daily activities during the three-week study period and did not receive ocular physiotherapy, ocular exercises, or a sham intervention.

The I-Exercises module consisted of seven sequential components shown in Table 1:

(1) Warm-up exercises involving gentle blinking and palming techniques. (2) Stretching exercises involving horizontal, vertical, and diagonal eye movements. (3) Accommodative exercises using alternating near- and distant-fixation tasks. (4) Focusing exercises involving controlled convergence and divergence activities. (5) Strengthening exercises using repetitive coordinated ocular movement patterns. (6) Exercises for constitutional improvement involving visual conditioning and ocular circulation enhancement activities. (7) Relaxation exercises, including palming and visual relaxation techniques.

**Table 1:** I-Exercises protocol

Step	Exercise Component	Description	Dosage / Duration	Purpose
1	Warm-up Exercises	Sunning (Surya Chakshu Shyam Adhipati)	3 minutes	Promotes ocular relaxation and prepares the visual system for exercise.
		Blinking Exercise	10 repetitions	Improves tear film distribution, blinking efficiency, and reduces ocular surface fatigue.
		Tibetan Eye Chart	Clockwise and anticlockwise eye movements followed by diagonal movements; each sequence repeated 5 times	Improves ocular motility, visual tracking, and extraocular muscle coordination.
		Imaginary Eye Exercise	Right eye ×3, Left eye ×3, Both eyes ×3	Enhances ocular coordination, fixation, concentration, and visual tracking.
2	Stretching Exercises	Horizontal eye movements	10 repetitions (3-second hold)	Improves the flexibility and coordination of the extraocular muscles.
		Vertical eye movements	10 repetitions (3-second hold)	Improves ocular mobility and muscle flexibility.
		Diagonal eye movements	10 repetitions (3-second hold)	Enhances coordinated ocular movements in diagonal gaze.
		Circular eye movements	5 clockwise + 5 anticlockwise repetitions	Improves multidirectional ocular mobility and coordination.
3	Accommodative Exercise	Pencil Push-up Exercise	10 repetitions	Improves accommodative flexibility, convergence, and near focusing ability.
4	Focusing Exercises (Trataka Yoga)	Bahiranga Trataka (External Gazing)	5–10 minutes	Improves sustained visual fixation, concentration, and visual attention.
		Antaranga Trataka (Internal Gazing)	5–10 minutes	Enhances internal visual focus, concentration, and mental relaxation.
5	Strengthening Exercises	Resisted lateral eye movement	5 repetitions	Strengthens extraocular muscles and improves neuromuscular coordination.
		Resisted medial eye movement	5 repetitions each side	Improves medial ocular muscle strength and convergence control.
		Forceful eye closure and opening	10 repetitions	Activates eyelid musculature and improves ocular endurance.
		Cross-eye convergence exercise	5 repetitions	Enhances convergence ability and ocular muscle control.
6	Exercises for Constitutional Improvement	Cross-crawling with Figure-of-Eight Eye Tracking	10 repetitions (5 each side)	Improves bilateral coordination, visual-motor integration, and body-eye coordination.
7	Relaxation Exercises	Upadhyay Mudra	5 repetitions	Promotes physical and mental relaxation through controlled breathing and coordinated movements.
		Chinese Acupoint Massage	30–60 seconds per acupoint	Improves periocular circulation and reduces ocular fatigue.

The exercises were administered under supervision, five sessions per week for three weeks. Each session lasted approximately 30-40 minutes and was performed in a comfortable seated position under standardized lighting conditions. The intervention sessions were conducted in the Physiotherapy Department under the supervision of a qualified physiotherapist trained in ocular exercise protocols.

Outcome measure

Ocular fatigue was assessed using an adapted version of a previously published ocular fatigue questionnaire [10,15]. The questionnaire was adapted from the 12-item ocular fatigue symptom questionnaire described by Gupta and Aparna [10]. The symptoms assessed included tired eyes, sore/aching eyes, red eyes, irritated eyes, watery eyes, dry eyes, eyestrain, hot/burning eyes, blurred vision, difficulty focusing, double vision, and visual discomfort.

Each symptom was rated independently on a seven-point Likert scale ranging from 0 to 6, with higher scores indicating greater severity of the reported ocular fatigue symptom. The questionnaire was administered at baseline before the intervention and again after completion of the three-week intervention period in both groups.

The severity interpretation used for analysis was: 0 = none, 1-2 = mild, 3-4 = moderate, and 5-6 = severe.

Sample size

The sample size was determined based on an anticipated standardized effect size of 0.90, assuming a two-sided significance level (α) of 0.05 and a statistical power of 80%. The anticipated effect size was selected conservatively based on previous literature demonstrating a substantial reduction in eye-fatigue symptoms following ocular exercise interventions. Based on these assumptions, the required sample size was estimated using an appropriate statistical sample-size calculation. Considering the feasibility of recruitment and the exploratory nature of the study, a total of 20 participants were included in the study.

Data collection procedure

Demographic data and ocular fatigue scores were collected before randomization and initiation of the intervention. The same questionnaire was administered at the conclusion of the three-week intervention period. All participants completed the questionnaire at both assessment points, and no missing responses were reported.

Statistical analysis

Data entry and validation were performed in Microsoft Excel (Microsoft Corp., Redmond, WA, USA), and statistical calculations were performed in SPSS version 24 (IBM Corp., Armonk, NY, USA). Data were analyzed using appropriate statistical methods based on the distribution and type of the variables. Ocular fatigue symptoms were assessed at baseline and after completion of the intervention in both groups. Within-group and between-group comparisons were performed using the appropriate statistical tests.

Individual ocular fatigue symptoms were considered symptom-level exploratory outcomes. Because multiple symptom-level comparisons were performed, no formal adjustment for multiplicity was applied. Therefore, individual symptom-level p-values were interpreted cautiously, and these findings were considered exploratory and hypothesis-generating. A p-value < 0.05 was considered statistically significant.

## Results

Table 2 presents the baseline demographic characteristics of the study participants. The mean age was 21.15 ± 2.04 years in the experimental group and 20.82 ± 1.97 years in the control group, with an overall mean age of 20.99 ± 2.00 years. The sex distribution was comparable between the two groups, with males comprising 40.0% of the experimental group and 35.0% of the control group. Females comprised 60.0% and 65.0% of the experimental and control groups, respectively.

**Table 2:** Demographic details

Characteristic	Experimental (n = 20)	Control (n = 20)	Total (N = 40)
Age (years), mean ± SD	21.15 ± 2.04	20.82 ± 1.97	20.99 ± 2.00
Sex, n (%)			
Male	8 (40.0%)	7 (35.0%)	15 (37.5%)
Female	12 (60.0%)	13 (65.0%)	25 (62.5%)

Baseline ocular fatigue scores

Baseline assessment demonstrated that participants experienced predominantly mild-to-moderate ocular fatigue symptoms prior to the intervention. Higher baseline mean scores were observed for tired eyes, watery eyes, eye strain, blurred vision, and difficulty focusing, whereas lower scores were noted for red eyes, dry eyes, and burning eyes. The severity distribution further showed that the majority of participants were classified within the moderate severity category for most symptoms, confirming a comparable baseline ocular fatigue profile before intervention. Severity categories were defined as none (0), mild (1-2), moderate (3-4), and severe (5-6). No participant recorded a score of 6; therefore, 5 represented the highest observed severity score in the present study (Table 3).

**Table 3:** Baseline Ocular Fatigue Scores and Symptom Severity Distribution of Participants (N = 40) Values are presented as mean ± standard deviation (SD) together with the frequency (n) and percentage (%) of participants within each symptom severity category. Severity categories were derived from the ocular fatigue questionnaire scores (None = 0, Mild = 1–2, Moderate = 3–4, Severe = 5-6). No participant recorded a score of 6; therefore, 5 was the highest observed score in the present study. Higher scores indicate greater ocular fatigue severity.

Symptom	Mean ± SD	None n (%)	Mild n (%)	Moderate n (%)	Severe n (%)	Minimum	Maximum
Tired eyes	3.63 ± 0.84	–	4 (10.0)	31 (77.5)	5 (12.5)	2	5
Sore/aching eyes	3.03 ± 0.77	–	10 (25.0)	29 (72.5)	1 (2.5)	2	5
Red eyes	0.45 ± 0.64	25 (62.5)	15 (37.5)	–	–	0	2
Irritated eyes	1.28 ± 0.78	7 (17.5)	32 (80.0)	1 (2.5)	–	0	3
Watery eyes	3.33 ± 0.73	–	5 (12.5)	34 (85.0)	1 (2.5)	2	5
Dry eyes	0.98 ± 1.00	17 (42.5)	20 (50.0)	3 (7.5)	–	0	3
Eye strain	3.20 ± 1.20	2 (5.0)	6 (15.0)	27 (67.5)	5 (12.5)	0	5
Burning eyes	0.85 ± 1.03	20 (50.0)	16 (40.0)	4 (10.0)	–	0	3
Blurred vision	3.80 ± 1.38	2 (5.0)	4 (10.0)	20 (50.0)	14 (35.0)	0	5
Difficulty focusing	2.73 ± 1.30	2 (5.0)	20 (50.0)	14 (35.0)	4 (10.0)	0	5
Vision discomfort	2.50 ± 1.34	4 (10.0)	14 (35.0)	20 (50.0)	2 (5.0)	0	5

Within-group comparison in the experimental group

Following completion of the three-week I-Exercises intervention, participants in the experimental group demonstrated statistically significant reductions in ocular fatigue scores across all assessed symptoms. Significant improvements were observed for tired eyes, sore/aching eyes, red eyes, irritated eyes, watery eyes, dry eyes, eye strain, burning eyes, blurred vision, difficulty focusing, and vision discomfort (all p < 0.05), indicating a substantial reduction in ocular fatigue following the intervention (Table 4).

**Table 4:** Pre- and Post-Intervention Comparison of Ocular Fatigue Scores in the Experimental Group (n = 20) Values are presented as mean ± standard deviation (SD) together with the frequency (n) and percentage (%) of participants within each symptom severity category. Severity categories were defined as None = 0, Mild = 1–2, Moderate = 3–4, and Severe = 5-6. No participant recorded a score of 6; therefore, 5 was the highest observed score in the present study. Within-group comparisons were performed using the Wilcoxon Signed Rank Test, with statistical significance set at p < 0.05.

Symptom	Pre (Mean ± SD)	Pre Severity Distribution, n (%)	Post (Mean ± SD)	Post Severity Distribution, n (%)	Z value	p value
Tired eyes	3.75 ± 0.79	Mild: 1 (5.0), Moderate: 16 (80.0), Severe: 3 (15.0)	1.90 ± 0.85	None: 1 (5.0), Mild: 14 (70.0), Moderate: 5 (25.0)	−4.058	<0.001
Sore/aching eyes	3.15 ± 0.88	Mild: 5 (25.0), Moderate: 14 (70.0), Severe: 1 (5.0)	1.10 ± 0.64	None: 3 (15.0), Mild: 17 (85.0)	−4.128	<0.001
Red eyes	0.50 ± 0.69	None: 12 (60.0), Mild: 8 (40.0)	0.25 ± 0.55	None: 16 (80.0), Mild: 4 (20.0)	−2.236	0.025
Irritated eyes	1.30 ± 0.86	None: 4 (20.0), Mild: 15 (75.0), Moderate: 1 (5.0)	0.25 ± 0.55	None: 16 (80.0), Mild: 4 (20.0)	−3.520	<0.001
Watery eyes	3.45 ± 0.83	Mild: 3 (15.0), Moderate: 16 (80.0), Severe: 1 (5.0)	1.80 ± 0.89	None: 1 (5.0), Mild: 16 (80.0), Moderate: 3 (15.0)	−3.598	<0.001
Dry eyes	1.00 ± 0.97	None: 8 (40.0), Mild: 11 (55.0), Moderate: 1 (5.0)	0.25 ± 0.44	None: 15 (75.0), Mild: 5 (25.0)	−3.217	0.001
Eye strain	3.35 ± 1.14	None: 1 (5.0), Mild: 2 (10.0), Moderate: 15 (75.0), Severe: 2 (10.0)	0.55 ± 0.60	None: 10 (50.0), Mild: 10 (50.0)	−3.901	<0.001
Burning eyes	0.75 ± 0.91	None: 10 (50.0), Mild: 9 (45.0), Moderate: 1 (5.0)	0.20 ± 0.41	None: 16 (80.0), Mild: 4 (20.0)	−3.051	0.002
Blurred vision	4.00 ± 1.38	None: 1 (5.0), Mild: 3 (15.0), Moderate: 7 (35.0), Severe: 9 (45.0)	1.90 ± 1.33	None: 2 (10.0), Mild: 14 (70.0), Moderate: 2 (10.0), Severe: 2 (10.0)	−3.714	<0.001
Difficulty focusing	3.00 ± 1.41	None: 1 (5.0), Mild: 8 (40.0), Moderate: 8 (40.0), Severe: 3 (15.0)	0.90 ± 0.72	None: 6 (30.0), Mild: 14 (70.0)	−3.861	<0.001
Vision discomfort	2.45 ± 1.28	None: 1 (5.0), Mild: 9 (45.0), Moderate: 9 (45.0), Severe: 1 (5.0)	0.30 ± 0.47	None: 14 (70.0), Mild: 6 (30.0)	−3.859	<0.001

Within-group comparison in the control group

The control group demonstrated no statistically significant improvement in ocular fatigue over the study period. Although watery eyes showed a marginal reduction (p = 0.046), all remaining symptoms remained statistically unchanged, indicating minimal spontaneous improvement without intervention (Table 5).

**Table 5:** Pre- and Post-Intervention Comparison of Ocular Fatigue Scores in the Control Group (n = 20) Values are presented as mean ± standard deviation (SD) together with the frequency (n) and percentage (%) of participants within each symptom severity category. Severity categories were defined as None = 0, Mild = 1–2, Moderate = 3–4, and Severe = 5. Within-group comparisons were performed using the Wilcoxon Signed Rank Test, with statistical significance set at p < 0.05.

Symptom	Pre (Mean ± SD)	Pre Severity Distribution, n (%)	Post (Mean ± SD)	Post Severity Distribution, n (%)	Z value	p value
Tired eyes	3.50 ± 0.89	Mild: 3 (15.0), Moderate: 15 (75.0), Severe: 2 (10.0)	3.40 ± 0.75	Mild: 2 (10.0), Moderate: 17 (85.0), Severe: 1 (5.0)	−0.816	0.414
Sore/aching eyes	2.90 ± 0.64	Mild: 5 (25.0), Moderate: 15 (75.0)	2.90 ± 0.55	Mild: 4 (20.0), Moderate: 16 (80.0)	0.000	1.000
Red eyes	0.40 ± 0.60	None: 13 (65.0), Mild: 7 (35.0)	0.45 ± 0.60	None: 12 (60.0), Mild: 8 (40.0)	−1.000	0.317
Irritated eyes	1.25 ± 0.72	None: 3 (15.0), Mild: 17 (85.0)	1.10 ± 0.72	None: 4 (20.0), Mild: 16 (80.0)	−0.905	0.366
Watery eyes	3.20 ± 0.62	Mild: 2 (10.0), Moderate: 18 (90.0)	3.00 ± 0.73	Mild: 5 (25.0), Moderate: 15 (75.0)	−2.000	0.046
Dry eyes	0.95 ± 1.05	None: 9 (45.0), Mild: 9 (45.0), Moderate: 2 (10.0)	0.75 ± 0.97	None: 11 (55.0), Mild: 8 (40.0), Moderate: 1 (5.0)	−1.633	0.102
Eye strain	3.05 ± 1.28	None: 1 (5.0), Mild: 4 (20.0), Moderate: 12 (60.0), Severe: 3 (15.0)	2.95 ± 1.19	None: 1 (5.0), Mild: 5 (25.0), Moderate: 13 (65.0), Severe: 1 (5.0)	−0.816	0.414
Burning eyes	0.95 ± 1.15	None: 10 (50.0), Mild: 7 (35.0), Moderate: 3 (15.0)	0.80 ± 1.15	None: 12 (60.0), Mild: 5 (25.0), Moderate: 3 (15.0)	−1.732	0.083
Blurred vision	3.60 ± 1.39	None: 1 (5.0), Mild: 3 (15.0), Moderate: 11 (55.0), Severe: 5 (25.0)	3.60 ± 1.39	None: 1 (5.0), Mild: 3 (15.0), Moderate: 11 (55.0), Severe: 5 (25.0)	0.000	1.000
Difficulty focusing	2.45 ± 1.15	None: 1 (5.0), Mild: 12 (60.0), Moderate: 6 (30.0), Severe: 1 (5.0)	2.45 ± 1.19	None: 1 (5.0), Mild: 11 (55.0), Moderate: 7 (35.0), Severe: 1 (5.0)	0.000	1.000
Vision discomfort	2.55 ± 1.43	None: 3 (15.0), Mild: 5 (25.0), Moderate: 11 (55.0), Severe: 1 (5.0)	2.60 ± 1.35	None: 2 (10.0), Mild: 6 (30.0), Moderate: 11 (55.0), Severe: 1 (5.0)	−0.577	0.564

Between-group comparison of post-intervention scores

Post-intervention comparison demonstrated significantly lower ocular fatigue symptom scores in the experimental group than in the control group for tired eyes, sore/aching eyes, irritated eyes, watery eyes, eyestrain, blurred vision, difficulty focusing, and visual discomfort. No statistically significant between-group differences were observed for red eyes, dry eyes, hot/burning eyes, or double vision. These findings indicate that the Comprehensive Ocular Exercise Module (I-Exercises) was associated with significant improvement in several ocular fatigue symptoms compared with no intervention (Table 6).

**Table 6:** Post-Intervention Comparison of Ocular Fatigue Scores Between the Experimental (n = 20) and Control (n = 20) Groups Values are based on the Mann–Whitney U test for comparison between the experimental and control groups. *p < 0.05 was considered statistically significant.

Ocular fatigue symptom	Experimental Mean Rank	Control Mean Rank	Mann–Whitney U	Z	p-value
Tired eyes	12.58	28.43	41.50	-4.453	<0.001*
Sore/aching eyes	11.00	30.00	10.00	-5.362	<0.001*
Red eyes	18.60	22.40	162.00	-1.283	0.199
Irritated eyes	14.30	26.70	76.00	-3.669	<0.001*
Watery eyes	13.63	27.38	62.50	-3.890	<0.001*
Dry eyes	17.88	23.13	147.50	-1.681	0.093
Eye strain	11.63	29.38	22.50	-4.917	<0.001*
Burning eyes	18.00	23.00	150.00	-1.676	0.094
Blurred vision	14.45	26.55	79.00	-3.338	0.001*
Difficulty focusing	13.15	27.85	53.00	-4.118	<0.001*
Double vision	18.83	22.18	166.50	-1.157	0.247
Visual discomfort	12.10	28.90	32.00	-4.735	<0.001*

## Discussion

The present randomized controlled trial evaluated the effectiveness of the Comprehensive Ocular Exercise Module (I-Exercises) in reducing ocular fatigue symptoms among students with myopia. Compared with the control group, participants in the experimental group demonstrated significant improvement in several self-reported ocular fatigue symptoms after three weeks of intervention. These findings provide preliminary evidence that structured ocular exercises may help reduce symptoms associated with prolonged near-vision activities and digital device use.

Both groups showed primarily mild-to-moderate levels of ocular fatigue at baseline, particularly with symptoms such as tired eyes, eyestrain, blurred vision, and difficulty focusing. This pattern is consistent with previous studies reporting a high prevalence of digital eye strain among college-aged individuals, particularly in association with prolonged near work and increased screen exposure [16,17]. The similarity in baseline ocular fatigue between the groups also supports their comparability and provides a basis for interpreting the post-intervention differences.

Several ocular fatigue symptoms, including tired eyes, sore/aching eyes, eyestrain, blurred vision, difficulty focusing, and visual discomfort, showed significant improvement in the experimental group. Between-group analysis further demonstrated significantly lower post-intervention symptom scores in the experimental group for tired eyes, sore/aching eyes, irritated eyes, watery eyes, eyestrain, blurred vision, difficulty focusing, and visual discomfort. In contrast, red eyes, dry eyes, hot/burning eyes, and double vision did not demonstrate statistically significant between-group differences. These findings are generally consistent with previous research suggesting that ocular and yoga-based eye exercises may have beneficial effects on symptoms of visual fatigue [18]. The I-Exercises module incorporates relaxation, strengthening, accommodative, and focused eye movements, which may collectively reduce functional visual stress and improve visual comfort during prolonged near-vision activities. However, the specific physiological mechanisms responsible for the observed improvements were not directly evaluated in the present study and therefore remain potential explanations rather than confirmed mechanisms.

Prolonged near work and digital screen exposure have been associated with accommodative stress, vergence changes, altered accommodative responses, and symptoms such as blurred vision and difficulty focusing [8,19,20]. Sustained fixation may also increase oculomotor and muscular demands, contributing to visual fatigue [21,22]. The controlled eye movements and relaxation components incorporated into I-Exercises may potentially help reduce this functional demand and promote more comfortable visual performance during prolonged near tasks. In addition, blinking and relaxation exercises included in the module may support ocular surface comfort by reducing symptoms associated with tear-film instability and reduced blinking during screen use [23].

The improvement in symptoms observed in the present study may therefore reflect a combination of relaxation, controlled ocular movements, and reduced visual stress during sustained near activities. Similar mechanisms have been proposed in previous literature, in which ocular exercises were associated with improved visual comfort and reduced symptoms of eye fatigue [20-23]. Importantly, however, the present study did not directly measure accommodation, vergence, oculomotor coordination, extraocular muscle activity, or ocular surface parameters. Therefore, these mechanisms cannot be confirmed from the current findings. The intervention should also be considered an approach for reducing ocular fatigue symptoms rather than modifying refractive error itself, which is consistent with the distinction between myopia and symptoms of visual fatigue [8,23,24].

The between-group findings provide preliminary support for a potential beneficial effect of I-Exercises on several ocular fatigue symptoms. The randomized controlled design strengthens this interpretation by providing a comparison group that did not receive the intervention. However, because the control group received neither an active nor a sham intervention, factors such as participant expectations, attention received during the intervention, and other behavioral changes cannot be completely excluded. Therefore, the findings should be interpreted as preliminary evidence of improvement in self-reported ocular fatigue symptoms rather than definitive evidence of clinical effectiveness.

Because multiple individual ocular fatigue symptoms were assessed and analyzed, the possibility of type I error due to multiple comparisons must also be considered. No formal adjustment for multiplicity was applied to these symptom-level analyses. Accordingly, the individual symptom findings should be interpreted cautiously as exploratory and hypothesis-generating rather than as independent confirmatory evidence of treatment efficacy. The inclusion of both statistically significant and nonsignificant symptom-level findings in the between-group analysis provides a more complete representation of the observed effects and reduces the potential for selective outcome reporting.

There are several limitations to the present investigation. The intervention was conducted over three weeks without long-term follow-up, and the sample size was small (n = 40). The study was designed as a preliminary randomized controlled trial and no formal power calculation based on a predefined primary outcome was performed. Therefore, the findings should be considered preliminary and require confirmation in larger adequately powered studies. Future research with larger sample sizes and longer follow-up periods may provide further information regarding the persistence and generalizability of the observed effects.

The study primarily relied on a self-reported questionnaire to assess ocular fatigue. Although the questionnaire was adapted from a previously published ocular fatigue symptom questionnaire [10], subjective assessment may be influenced by participant expectations and reporting behavior. Incorporating objective ophthalmic measurements, such as accommodation, vergence, ocular motility, visual performance, and ocular surface parameters, may provide additional information regarding the effects of I-Exercises and help clarify the mechanisms underlying symptom improvement.

The results may not be directly generalizable to other age groups or populations because the study included only young adults with myopia aged 18-25 years. Furthermore, neither an active nor a sham intervention was provided to the control group. The possible influence of participant attention and expectations therefore cannot be completely excluded. Because of the nature of the intervention, blinding of participants and therapists was not feasible. In addition, screen exposure was not formally monitored during the trial and may have influenced ocular fatigue symptoms. Future controlled studies should therefore consider active or sham control conditions, longer follow-up, objective outcome measures, and monitoring of relevant behavioral factors such as screen exposure.

Despite these limitations, the present study provides preliminary clinical evidence regarding the potential contribution of the I-Exercises Comprehensive Ocular Exercise Module to reducing self-reported ocular fatigue symptoms among students with myopia. The findings build upon the earlier development and standardization of the I-Exercises protocol through a Delphi-based process [14]. Larger, adequately powered randomized controlled trials with longer intervention and follow-up periods, objective ophthalmic outcome measures, and active or sham control groups are warranted to confirm these findings and better understand the underlying mechanisms.

Overall, the present findings suggest that I-Exercises may be a potentially useful, non-invasive approach for reducing selected symptoms of ocular fatigue in young adults with myopia. However, the findings should be interpreted cautiously because of the small sample size, short intervention period, subjective outcome assessment, absence of long-term follow-up, and lack of adjustment for multiple symptom-level comparisons. Further research is required to establish the long-term effectiveness, clinical relevance, and broader applicability of the intervention.

## Conclusions

The present randomized controlled trial provides preliminary evidence that the Comprehensive Ocular Exercise Module (I-Exercises) may help reduce selected self-reported ocular fatigue symptoms among young adults with myopia. The observed improvements following the intervention support the potential use of structured ocular exercises as a simple, non-invasive approach for reducing symptoms of ocular fatigue in this population. However, the findings should be interpreted cautiously in view of the small sample size, short intervention period, subjective outcome assessment, absence of an active or sham control group, and the exploratory nature of the individual symptom-level analyses. Larger, adequately powered randomized controlled trials incorporating objective ocular measurements, appropriate control conditions, and longer follow-up are warranted to confirm the effectiveness and clinical applicability of the I-Exercises module.

## Appendices

**Table 7:** Ocular Fatigue Questionnaire Severity interpretation: 0 = None; 1–2 = Mild; 3–4 = Moderate; 5–6 = Severe. Definitions: None – symptom absent. Mild – symptom present with little discomfort or interference. Moderate – noticeable symptom with moderate discomfort or interference. Severe – prominent symptom with substantial discomfort or interference with usual visual activities. Scoring: Each symptom is rated independently from 0 to 6, with higher scores indicating greater severity of the reported symptom.

Ocular fatigue symptom	0	1	2	3	4	5	6
Tired eyes	☐	☐	☐	☐	☐	☐	☐
Sore/aching eyes	☐	☐	☐	☐	☐	☐	☐
Red eyes	☐	☐	☐	☐	☐	☐	☐
Irritated eyes	☐	☐	☐	☐	☐	☐	☐
Watery eyes	☐	☐	☐	☐	☐	☐	☐
Dry eyes	☐	☐	☐	☐	☐	☐	☐
Eyestrain	☐	☐	☐	☐	☐	☐	☐
Hot/burning eyes	☐	☐	☐	☐	☐	☐	☐
Blurred vision	☐	☐	☐	☐	☐	☐	☐
Difficulty focusing	☐	☐	☐	☐	☐	☐	☐
Double vision	☐	☐	☐	☐	☐	☐	☐
Vision discomfort	☐	☐	☐	☐	☐	☐	☐
